# A novel nucleo-cytoplasmic hybrid clone formed via androgenesis in polyploid gibel carp

**DOI:** 10.1186/1756-0500-4-82

**Published:** 2011-03-28

**Authors:** Zhong-Wei Wang, Hua-Ping Zhu, Da Wang, Fang-Fang Jiang, Wei Guo, Li Zhou, Jian-Fang Gui

**Affiliations:** 1 State Key Laboratory of Freshwater Ecology and Biotechnology, Institute of Hydrobiology, Chinese Academy of Sciences, Wuhan, 430072, China

## Abstract

**Background:**

Unisexual vertebrates have been demonstrated to reproduce by gynogenesis, hybridogenesis, parthenogenesis, or kleptogenesis, however, it is uncertain how the reproduction mode contributes to the clonal diversity. Recently, polyploid gibel carp has been revealed to possess coexisting dual modes of unisexual gynogenesis and sexual reproduction and to have numerous various clones. Using sexual reproduction mating between clone D female and clone A male and subsequent 7 generation multiplying of unisexual gynogenesis, we have created a novel clone strain with more than several hundred millions of individuals. Here, we attempt to identify genetic background of the novel clone and to explore the significant implication for clonal diversity contribution.

**Methods:**

Several nuclear genome markers and one cytoplasmic marker, the mitochondrial genome sequence, were used to identify the genetic organization of the randomly sampled individuals from different generations of the novel clone.

**Results:**

Chromosome number, *Cot*-1 repetitive DNA banded karyotype, microsatellite patterns, AFLP profiles and transferrin alleles uniformly indicated that nuclear genome of the novel clone is identical to that of clone A, and significantly different from that of clone D. However, the cytoplasmic marker, its complete mtDNA genome sequence, is same to that of clone D, and different from that of clone A.

**Conclusions:**

The present data indicate that the novel clone is a nucleo-cytoplasmic hybrid between the known clones A and D, because it originates from the offspring of gonochoristic sexual reproduction mating between clone D female and clone A male, and contains an entire nuclear genome from the paternal clone A and a mtDNA genome (cytoplasm) from the maternal clone D. It is suggested to arise via androgenesis by a mechanism of ploidy doubling of clone A sperm in clone D ooplasm through inhibiting the first mitotic division. Significantly, the selected nucleo-cytoplasmic hybrid female still maintains its gynogenetic ability. Based on the present and previous findings, we discuss the association of rapid genetic changes and high genetic diversity with various ploidy levels and multiple reproduction modes in several unisexual and sexual complexes of vertebrates and even other invertebrates.

## Background

Since Amazon molly *Poecilia formosa*, the first clonal reproduction vertebrate, was found in 1932[1], about 90 all-female unisexual complexes have been reported in fish, amphibians and reptiles[2]. These unisexual animals have been revealed to reproduce by gynogenesis, hybridogenesis, parthenogenesis, or kleptogenesis[3-6], but it has remained uncertain how the reproduction mode contributes to the clonal diversity. Recently, unique dual modes of unisexual gynogenesis and sexual reproduction have been discovered in polyploid gibel carp[7,8], which gives a significant clue to the formation of clone diversity in unisexual vertebrates.

Gibel carp, also commonly known as silver crucian carp or Prussian carp, which was previously nominated as a subspecies (*Carassius auratus gibelio*) of goldfish, has been currently suggested as a separate species *Carassius gibelio*[9]. It was preliminarily uncovered to have unisexual reproduction ability of allogynogenesis early in the last century[10,11]. Allogynogenesis is a form of gynogenesis stimulated by heterologous sperm from other fish species. In allogynogenesis, some supernumerary minichromosomes or chromosomal fragments can be accidently incorporated into the maternal genomes[12,13], but the heterologous sperm does not decondense and fuse with the female pronucleus[8,11]. Diverse karyotypes with 156 or 162 chromosomes[14] and triploid origin relative to goldfish with 100 chromosomes[15] were confirmed in wild gibel carp populations. Moreover, numerous different clones and genetic diversity were discriminated by serum transferrin phenotypes[16], RAPD and SCAR markers[17,18], microsatellite markers[19], transferrin allele polymorphism[20,21], and mtDNA control region sequences[22]. Significantly, an approximate 1%-10% of males identical to the females in genetic background were found in natural habitats, and genetic recombination evidence was obtained in the offspring produced by bisexual mating between different clones[7]. Therefore, genetic evidence indicates that gibel carp have coexisting dual modes of unisexual gynogenesis and gonochoristic sexual reproduction, as it can reproduce all-female offspring through unisexual gynogenesis when the eggs are activated by heterologous sperm from other fish species, and also produces female and male offspring through sexual reproduction when the eggs are inseminated by homologous sperm from the gibel carp males[8].

Theoretically, the dual reproduction modes provide a unique way for creating novel clones in laboratory, because numerous genetic recombination offspring can be obtained by sexual reproduction mating between different clones, and some better and valuable clones can be selected and proliferated by unisexual gynogenesis[8]. To exploit the new breeding potential, we performed numerous sexual mating experiments between different clones since 1996. Clone A and clone D are very diverse among the identified clones. Karyotype of clone A individuals contains 156 chromosomes, which are composed of 36 metacentric(m), 54 submetacentric(sm), 36 subtelocentric(st), 24 acrocentric(t), and six small chromosomes, whereas karyotype of clone D individuals has 162 chromosomes, with 42 m, 54 sm, 36 st, 24 t, and six small chromosomes[14]. Through sexual reproduction mating between clones A and D, we found a few of fast-growing individuals. Significantly, the fast-growing individuals still possess its unisexual reproduction ability of gynogenesis. Thereby, the novel clone A^+ ^was created by originally sexual mating between clone D female and clone A male, and rapidly multiplied up to several hundred millions by subsequent 7 generations of unisexual gynogenesis[8]. Here, we summarize formation process of the novel clone strain and attempt to identify the genetic organization and background by nuclear and cytoplasmic markers including chromosome number count, *Cot*-1 DNA fluorescent banding karyotype analysis, microsatellite electrophoretic pattern, AFLP profile, transferrin allele identification and mitochondrial genome sequence comparison, because most of them had been proven to be particularly valuable for clone discrimination, diversity evaluation and genealogical relationship analysis in several unisexual vertebrates, such as gynogenetic Amazon molly *Poecilia formosa*[23,24], gynogenetic *Phoxinus eos-neogaeus*[25], hybridogenetic *Poeciliopsis*[26-28], hybridogenetic water frog *Rana esculenta *[29], kleptogenetic salamanders[30-35], parthenogenetic lizards[36], and the gynogenetic gibel carp[19-22,37]. Based on these studies, we explore and discuss the significant implication for clonal diversity contribution.

## Methods

### Source of samples

Clone D female and clone A male were used as the maternal and paternal for the propagation experiments of gonochoristic sexual reproduction mating. Control gynogenetic individuals of clone D were inseminated by sperm from red common carp (*Cyprinus carpio*) to activate the eggs. As described previously, spawning was artificially induced by two intraperitoneal injections with a mixture of acetone-dried carp pituitary, hCG and LRH-A[38]. Ovulated eggs from clone D were divided into two parts and respectively inseminated with sperm from a clone A male and from a red common carp male. The produced offspring were respectively cultured in separate fishponds. After they reached to adults for one year, the phenotype, size and sex were determined. Subsequently, one fast-growing clone A-like individual was selected as the clonal maternal and its unisexual reproduction ability of gynogenesis was demonstrated by Xingguo red common carp (*Cyprinus carpio*) sperm stimulation, because the offspring are all-females, and identical to the maternal. Moreover, they have been proliferated by 7 successive generations of gynogenesis with Xingguo red common carp (*Cyprinus carpio*) sperm stimulation, and more than several hundred millions of clonal individuals have been produced. From the fifth generation, the clonal strain as a new aquatic variety has been introduced into more than 10 hatcheries, and their gynogenetic offspring were cultured throughout China. As a stock hatchery, the successively gynogenetic offspring of 7 generations have been maintained in the Guanqiao Experimental Station of the Hydrobiology Institute in Wuhan, Hubei Province, and all the samples for genetic analysis were collected from the Station. Besides the maternal and paternal, generally, ten individuals were randomly sampled from each analyzed clone and generation, and their fin tissues and blood cells were sampled for DNA extraction and DNA content measurement respectively. The fin tissues were stored in 95% ethanol at -20°C and blood cells were fixed by 70% ethanol and stored at 4°C.

### Genomic DNA extraction and *Cot*-1 DNA isolation

Genomic DNA were extracted individually from fin tissues as described previously[17]. *Cot*-1 DNA was isolated as described previously[39], and labeled with biotin-16-dUTP by a nick translation reaction for FISH.

### Chromosome preparation and fluorescence *in situ *hybridization

Chromosome metaphases were prepared from kidney cells of at least 5 individuals for each clone by the method of kidney cell-phytohemagglutinin (PHA) culture *in vivo *and then were counterstained with DAPI. Fluorescent *in situ *hybridization (FISH) of *Cot*-1 DNA was performed according to the method described previously[15], and images were acquired using a Leica inverted DMIRE2 microscope and a Leica LCS SP2 confocal image system (Leica, Germany).

### Genetic marker application

Ten microsatellite primers were applied to amplify microsatellites in genomic DNA and PCR amplification was performed as the described method[19]. As for AFLP analysis, genome DNA was digested with *EcoR *I and *Mse *I and then selective amplification was performed using ten primer combinations[37]. Products of microsatellite and AFLP were separated using 6% denaturing polyacrylamide electrophoresis and were visualized by silver staining[40]. Transferrin alleles were amplified using special primer (Tf760 and TF1162) and then were cloned and sequenced as described previously[20,21]. To obtain the complete mitochondrial genome, twenty conserved primers[41] were used to amplify contiguous and overlapping fragments. According to the aligned sequences, another two pairs of primers were designed to amplify the fragments including the varied sites for further validation.

### Sperm DNA content measurement

Clone A sperms were sampled from 8 individuals in reproduction season and 20 μl of them were respectively dropped into 1 ml of 70% ethanol. The fixed sperms at 4°C overnight were washed with PBS buffer for 3 times at 1000 rpm for 5 min. The pellets were resuspended in 0.5 ml of 0.5% pepsin in 0.1 M HCl. After the solution were incubated at room temperature for 30 min with gentle shaking, 100 μl of them was stained by adding 2 ml of propidium iodide solution (40 μgPI/ml) containing 4 kU/ml RNase (DNase-free ) at room temperature for 3 h in the dark. After the mixed cells were filtered through a special nylon mesh, the DNA content measurement was performed as described previously[42] by Phoenix Flow Systems (Beckman and Coulter). The blood cells of clone A were also sampled and detected from the same individuals as controls under the same conditions.

### Data analysis

Microsatellite and AFLP amplification bands in the gibel carp clones were evaluated based on the electrophoretic patterns and the genetic diversities were calculated by Arlequin 3.11 software[43]. The gene regions of mitochondrial genome were identified with the homologous regions of the complete mitochondrial DNA sequence of triploid hybrids of tetraploid and Japanese crucian carp (GenBank accession number: AY771781). All sequences of transferrin and mitochondrial DNA were aligned and exported with CLUSTAL X [44] and Mega 4.0 software [45].

## Results

### Formation process of novel clone A^+^

**Figure 1:**
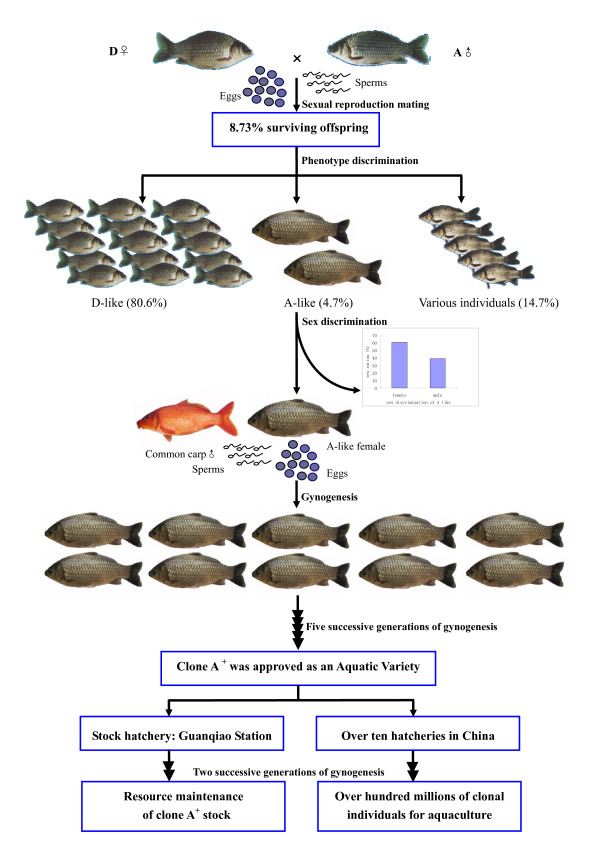
**A schematic diagram showing formation process of the novel clone A^+^**.

Figure 1 shows the schematic diagram of a novel clone strain formation through using dual modes of gonochoristic sexual reproduction and unisexual gynogenesis. Firstly, female clone D with 162 chromosomes was sexually mated with male clone A with 156 chromosomes, and the propagated offspring was strictly cultured in separate fishponds. In the gonochoristic sexual reproduction mating, only 8.73% of the fertilized eggs can develop into adults, and the surviving offspring display three different phenotypes. Most of them (over 80%) are identical to the maternal clone D, some (about 15%) differ from clone D and clone A, and a few of individuals (less than 5%) look like the paternal clone A. Significantly, the few individuals grow markedly faster than the maternal clone D and the paternal clone A, and there existed about 61% females and 39% males in them. Then, one individual of them was selected as the clonal maternal and proliferated by 7 successive generations of gynogenesis with Xingguo red common carp (*Cyprinus carpio*) sperm stimulation. Xingguo red common carp is a regional variety of common carp in Xingguo County, Jiangxi Province and has more than 1300 years of culture history. Owing to the significant growth superiority, the clone has been approved by National Certification Committee for Aquatic Varieties as a new aquatic variety that is suitable for aquaculture in China. From the fifth generation, the novel clonal variety has been introduced into more than 10 hatcheries, and their gynogenetic offspring were cultured throughout China. At present, more than several hundred millions of clonal individuals have been produced in the hatcheries. As a novel clone strain, it has been designated as clone A^+^, but its genetic organization and the mechanism of clone formation have been still unknown.

### Identical chromosome number and banded karyotype to clone A

**Figure 2:**
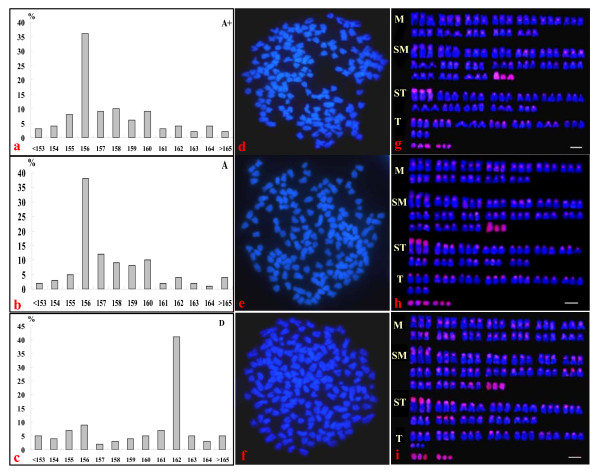
**Comparisons of chromosome number (a-c), representative metaphases (d-f) and triploid karyotypes of *Cot*-1 DNA fluorescent banding (g-i) among the novel clone A^+^(A^+^), clone A (A) and clone D (D)**.

We firstly performed chromosome number count and *Cot*-1 DNA fluorescent banding karyotype analysis of the clone A^+ ^metaphases sampled from the fifth generation, and compared with that of clone D and clone A. Figure 2 summarizes the data. In the clone A^+ ^(Figure 2a) and A (Figure 2b), 38% and 36% examined metaphases contain 156 chromosomes, whereas in clone D (Figure 2c), 41% counted metaphases possess 162 chromosomes. Therefore, the modal chromosome number and representative metaphase (Figure 2d, 2e) of clones A^+ ^are the same as those of clone A, which contain 156 chromosomes, whereas the modal chromosome number and representative metaphase of clone D (Figure 2f) possess 162 chromosomes.

Moreover, the *Cot*-1 repetitive DNA fluorescence banding shows homologous chromosome-specifically patterns, and non-homologous chromosomes have different banded patterns. According to the various fluorescent intensities that locate at centromeric regions and other interstitial regions and the differential chromosome size and shape, we compiled the triploid karyotypes of clones A^+^, clone A and clone D with three homologous chromosomes. In all the analyzed three karyotypes, there are 9 small chromosomes that are wholly labeled by the *Cot*-1 repetitive DNA fluorescence, in which three of them are submetacentric chromosomes, and others are six small chromosomes. Also, the short arms of three same size subtelocentric chromosomes are entirely stained by the *Cot*-1 repetitive DNA fluorescence in all examined three clones. Significantly, clones A^+ ^(Figure 2 g) and clone A (Figure 2 h) possess identical karyotypes that are comprised of 36 m, 54 sm, 36 st, 24 t, and six small chromosomes, whereas the karyotype of clone D (Figure 2i) is different from that of clones A^+ ^and clone A, which contains 42 m, 54 sm, 36 st, 24 t and six small chromosomes.

### Identical nuclear genome to clone A

**Figure 3:**
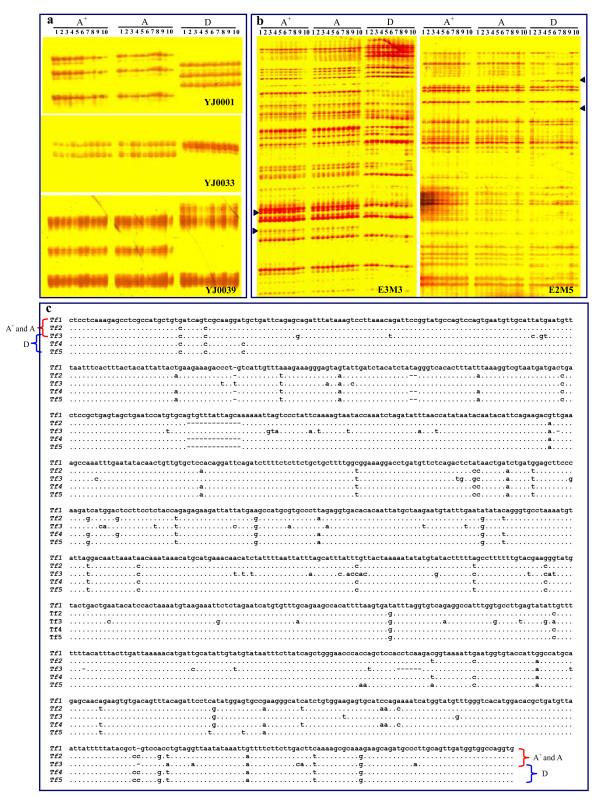
**Genetic discrimination of clone A^+^(A^+^), clone A (A) and clone D (D) through three different kinds of nuclear genome markers, such as microsatellite electrophoretic patterns (a), AFLP profiles (b) and transferrin allele sequences (c). **(a) Three typical microsatellite electrophoretic patterns amplified by the primer YJ0001, YJ0033 and YJ0039. (b) Two representative AFLP patterns amplified by the primer combinations E2M5 and E3M3. (c) The aligned five transferrin alleles *Tf1*, *Tf2*, *Tf3*, *Tf4 *and *Tf5 *identified from three clones.

Subsequently, the nuclear genome of clone A^+ ^was further discriminated by three nuclear genome markers, such as microsatellite electrophoretic pattern, AFLP profile, and transferrin allele identification. Firstly, ten pairs of microsatellite primers isolated previously[19] were used to assess genetic differences and similarities of clone A^+^, clone A and clone D (Table 1). In total, 27 microsatellite alleles were equally detected from clone A^+ ^and clone A, and 25 alleles were recognized from clone D. However, only three alleles were shared by clone A^+^, clone A and clone D from primer pairs 0039 and 0042. Figure 3a shows three typical microsatellite electrophoretic patterns amplified by primer pair YJ0001, YJ0033 and YJ0039, in which same allele patterns exist in clone A^+ ^and clone A, and various patterns appear in clone D. Additionally, completely identical allele patterns are observed among individuals within each clone because of clonal reproduction of gynogenesis.

**Table 1:** Primer pair sequences and allele distribution of 10 microsatellites among clone A, clone D and clone A^+ ^of gibel carp*

Locus (YJ)	Primer pair sequence	Annealing tempreture	Allele size (bp)		
			
			Clone A	Clone D	Clone A^+^
0001	5'-CTGGCATGAAGACTGGCTC-3' 5'-CAACAACACATATCAGCTCC-3'	53°C	84, 94, 100	88, 92, 96	84, 94, 100
0003	5'-TGAAGTTATTAGAAAGAGAG-3' 5'-CTTGATGATGTCTATGTGTG-3'	53°C	270, 288, 302	260, 290, 292	270, 288, 302
0004	5'-CATAGAGGCGTTTCATAGAG-3' 5'-CAGATAAATACAGTAAGCCA-3'	50 - 55°C	210, 220, 224	218, 222, 226	210, 220, 224
0005	5'-TAATAAGGTACATAGTCATAG-3' 5'-GTCAGCCTCCACCACGAATC-3'	50 - 55°C	226, 230, 234	222	226, 230, 234
0033	5'-CGGACACAAGAACGCCAAC-3' 5'-GGACTGGGCTGAAACTGATG-3'	50 - 55°C	172, 178	176	172, 178
0039	5'-GAAGAATACTTTATGACTGAGG-3' 5'-GACCAAGACAGACAGCCCAG-3'	50 - 55°C	138, 150, 156	136, 138, 156	138, 150, 156
0042	5'-GGCCACCTACAGTATATGC-3' 5'-GAAAACCAGGACCGACATG-3'	50 - 55°C	110, 114, 116	110, 112, 118	110, 114, 116
0040	5'-CCAGTATTAGGGAGCGTTC-3' 5'-GTTTCGTCTTCACAATCAGAA-3'	50 - 55°C	124, 138	132, 148	124, 138
0010	5'-GATGGTTGTGCTGTGAGCT-3' 5'-GAGTTCGTTTACATCTGGAC-3'	53°C	150, 156, 166	152, 160, 162	150, 156, 166
0022	5'-CACCAACTTTAGGCACATTTG-3' 5'-CCAGACTCCCACGTCATG-3'	53°C	144, 156	140, 162, 170	144, 156
Total	10		27	25	27

*For each clone and each generation, 10 individuals were sampled and analyzed, and clone A^+ ^samples of three generations, such as the third, fifth and seventh generation, were examined. No any individual difference was detected within each clone and among different generations.

AFLP profiles produced by 10 primer combinations provided much more abundant genetic information than that of the above microsatellites, and each primer combination amplified an average of 76.5 bands, ranging from 64 to 86 bands (Table 2). As shown in Figure 3b, completely identical AFLP profiles are observed in the sampled individuals of clone A^+ ^and clone A. However, the AFLP profiles of clone D are significantly different from that of clone A^+ ^and clone A. The percentages of the shared bands among three clones range from 66.2% to 82.7%, but 79 clone A^+ ^and clone A-specific bands and 88 clone D- specific bands are respectively detected from the amplified 10 AFLP profiles. The data again indicate that clone A^+ ^nuclear genome is same to clone A, and different from clone D.

**Table 2:** Comparison of amplification bands by AFLP among clone A, clone D and clone A^+ ^of gibel carp*

		No. of amplified bands					No. of clone-specific bands	
				
**No**.	AFLP primers	A	A^+^	D	total	No. of shared bands	A and A^+^	D
1	E-ACA M-CAG	62	62	67	75	54	8	13
2	E-AGG M-CTT	70	70	69	83	56	14	13
3	E-AAC M-CTG	61	61	62	74	49	12	13
4	E-AAG M-CTG	63	63	59	71	51	12	8
5	E-ACT M-CAT	71	71	77	81	67	4	10
6	E-ACG M-CTC	69	69	64	77	56	13	8
7	E-AAC M-CTA	66	66	71	75	62	4	9
8	E-AAG M-CAA	81	81	80	85	76	5	4
9	E-AAG M-CTA	59	59	57	64	52	7	5
10	E-ACA M-CTA	75	75	71	80	66	9	5
total		677	677	677	765	589	79	88

*For each clone and each generation, 10 individuals were sampled and analyzed, and clone A^+ ^samples of three generations, such as the third, fifth and seventh generation, were examined. No any individual difference was detected within each clone and among different generations.

Multiple ancient allelic lineages of transferrin had been identified in gibel carp[20,21]. To clone and identify all alleles of trnasferrin from the three clones, a pair of primers *Tf*760 (CTCCTCAAAGAGCCTCGC) and *Tf*1162 (CAAGGGCATCTGCTTCCT) was designed according to transferrin cDNA sequences (GenBank accession numbers: AY045574, AF457150, AF457151, AF518744, AF518745, AY323916) of gibel carp because high polymorphism sites had been revealed within this fragment among various transferrin alleles. Using this primer pair, we amplified and identified five different transferrin alleles (named *Tf1*, *Tf2*, *Tf3*, *Tf4 *and *Tf5*) from the three gibel carp clones (GenBank accession numbers: JF496199-JF496203). *Tf2*, *Tf4 *and *Tf5 *are 1170 bp in length, while the length of *Tf1 *and *Tf3 *are 1184 bp and 1177 bp respectively. From the aligned sequences, various mutations including deletion, transition and transversion are found among the five alleles (Figure 3c). Significantly, *Tf1 *and *Tf2 *appear specifically in clone A^+ ^and clone A, whereas *Tf4 *and *Tf5 *exist specifically in clone D. Only *Tf3 *is shared by all of the three clones. The divergence between *Tf3 *and the other alleles is from 0.080 to 0.087, and it is much higher than that of the other alleles (Table 3).

**Table 3:** Genetic divergences among the five transferrin alleles

	*Tf1*	*Tf2*	*Tf3*	*Tf4*	*Tf5*
*Tf1*	0.000	0.041	0.087	0.042	0.040
*Tf2*	0.041	0.000	0.080	0.001	0.011
*Tf3*	0.087	0.080	0.000	0.081	0.081
*Tf4*	0.042	0.001	0.081	0.000	0.010
*Tf5*	0.040	0.011	0.081	0.010	0.000

### Same mitochondrial genome to clone D

**Figure 4:**
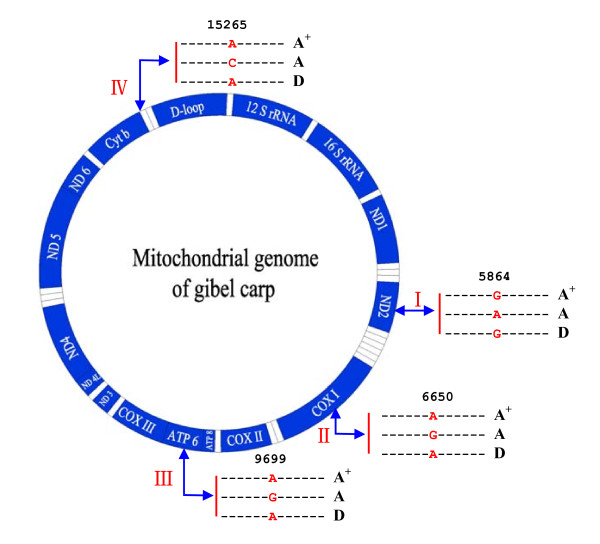
**Mitochondrial genome sequence comparison of clone A^+^(A^+^), clone A(A) and clone D(D)**. The complete genome sequences have been deposited in GenBank and the 4 single nucleotide polymorphisms are shown by the arrows at the corresponding positions.

Because clone A and clone D have been revealed from numerous haplotypes and diverse clones of gibel carp to belong to the same haplotype without any variation in the high polymorphism mtDNA control region sequences[22], therefore, we sequenced the complete mitochondrial genome sequences of clone A^+^, clone A and clone D using a PCR-based method. The complete mitochondrial genomes of all three clones are 16580 bp long (D-clone, JF496197; A clone JF496198), and contain the identical gene complement (13 protein-coding genes, 2 rRNA genes, 22 tRNA genes and a major non-coding D-loop control region) and gene order as found in most vertebrate mitochondrial genomes[46]. Only 4 single nucleotide polymorphisms are detected among the three complete mitochondrial genomes, and they exist respectively in ND2 gene (5864 nucleotide position), COX|gene (6650 nucleotide position), ATP6 gene (9699 nucleotide position) and a tRNA gene (15265 nucleotide position). Significantly, the 4 variation position nucleotides of clone A^+ ^are all identical to that of clone D, and all different from that of clone A (Figure 4). Moreover, we designed two pairs of variation position-specific primers according to the flanking region sequences at 5864 and 6650 nucleotide variation positions, and used them to amplify and sequence the mtDNA fragments from 10 different individuals of each clone. The sequencing data indicate that both of the variation nucleotides in all individuals of clone A^+ ^are same to that of clone D, and different from that of clone A, which further confirm the genetic consistency of mitochondrial genomes between clone A^+ ^and clone D.

### Genetic uniformity among different generations

**Figure 5:**
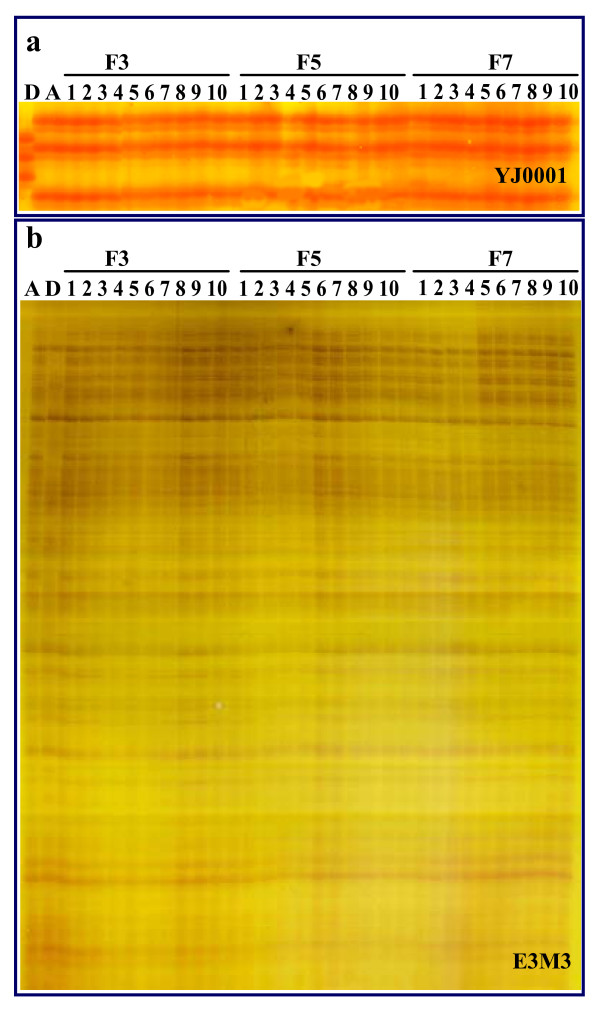
**Genetic uniformity among different generations of the clone A^+ ^and comparison with the maternal clone D and paternal clone A**. (a) One typical microsatellite electrophoretic pattern of different individuals sampled from the third(F3), fifth (F5) and seventh (F7) generations and the original maternal clone D (D) and paternal clone A (A) that were amplified by the primer YJ0001 in. (b) One representative AFLP pattern of different individuals sampled from the third (F3), fifth (F5) and seventh (F7) generations and the original maternal clone D (D) and paternal clone A (A) that were amplified by the primer combination E3M3.

To further confirm the above data and to judge the validity, we randomly sampled 10 individuals in different generations including the third generation (F_3_), the fifth generation (F_5_) and the seventh generation (F_7_) from the stock hatchery and comparatively analyzed the genetic profiles with the original maternal clone D and paternal clone A by nuclear markers of microsatellite and AFLP and cytoplasmic marker of mtDNA sequence. As shown in Figure 5, the genetic profiles of microsatellite (Figure 5a) and AFLP (Figure 5b) are highly identical among the samples collected from the three different generations, and are same to that of the paternal clone A, while are significantly different from that of clone D. Simultaneously, no any various nucleotides are detected by the two mtDNA fragment sequences among 30 individuals of the three generations, and their sequences are completely identical to that of clone D (data not shown). The additional data verify the genetic uniformity among different generations of the clone A^+ ^along with the seven generations.

### Clone A sperm DNA content

**Figure 6:**
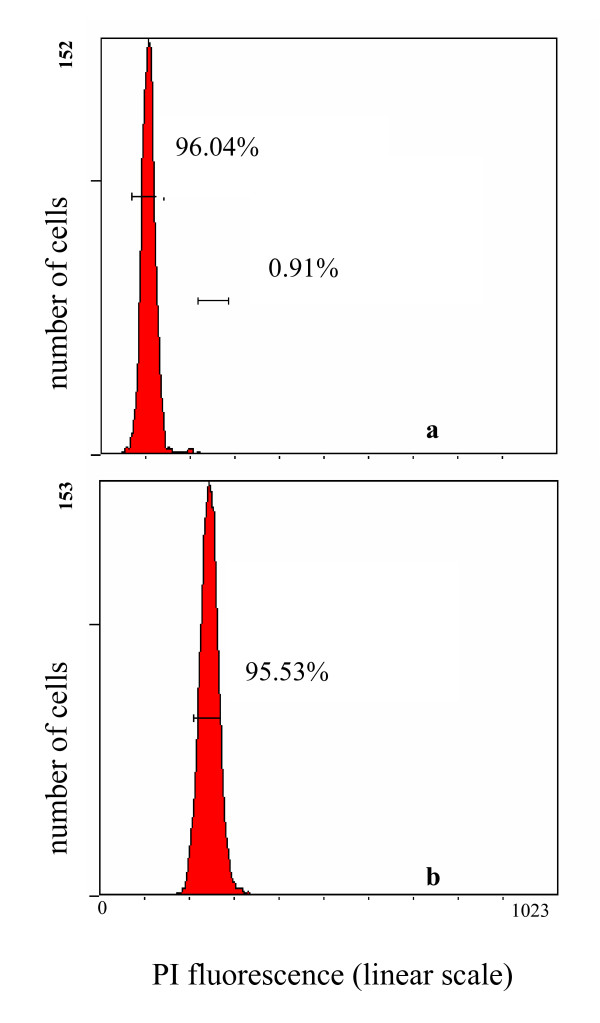
**Comparison of the DNA contents of sperms (a) and blood cells (b) of clone A**.

In comparison with only one peak of its own blood cells, clone A sperm DNA content measurement revealed one main peak and minor peak. As shown in Figure 6, the main peak fluorescence value is about half of the blood cells, whereas the minor peak value is basically same to that of the blood cells. Statistical data of 8 individuals indicate that the minor peak accounts for about 0.91% of the total sperms, implicating the existence of a few proportion of unreduced sperms.

## Discussion

In this study, we reported a detailed formation process of the novel clone strain, and analyzed and identified the genetic organization and background by nuclear and cytoplasmic genome markers. The current data clearly indicate that the nucleus of clone A^+ ^comes from that of clone A, because its chromosome number, *Cot*-1 repetitive DNA banded karyotype, and nuclear genome markers including microsatellite patterns, AFLP profiles and transferrin alleles are identical to that of clone A, and significantly different from that of clone D, whereas the cytoplasm comes from that of clone D, as the cytoplasmic marker-mtDNA genome is completely same to that of clone D, and different from that of clone A. Therefore, the major finding in this study is that the novel strain is clonal and represents a novel hybrid genotype with an entire nuclear genome from the paternal clone A and a mtDNA genome (cytoplasm) from the maternal clone D. And, the selected fast-growing individual as clonal maternal might be a nucleo-cytoplasmic hybrid between clone A and clone D, which might be formed via androgenesis of the clone A sperm in the clone D ooplasm.

As far as we know, androgenesis has been observed to occur naturally in interspecific hybrids of Sicilian stick insects[47,48] and triploid Asian clams (genus *Corbicula*)[49,50]. In fish, natural androgenesis has never been reported, but viable androgenetic fishes have been artificially induced in some ancient polyploid species, such as rainbow trout[51], common carp[52], loach[53,54], and sturgeon[55], and in a artificial allotetraploid hybrid of common carp and red crucian carp[41]. Generally, androgenesis involves loss or inactivation of the female genome and doubling of the paternal genome[56]. In the sexual mating between clone D female and clone A male, most of the fertilized eggs might undergo the fusion of clone D female pronucleus and clone A male pronucleus, and resulted in high mortality during embryo hatching and fingerling cultivation owing to the significant difference of chromosome number and karyotypes between them. The surviving offspring differ from clone D and clone A in morphological phenotype. Indeed, fusion failure of the female and male pronuclei might also occur in a few of the fertilized eggs, in which some developed into clone D-identical individuals, some developed into the clone A-like individuals. The clone D-identical individual might occur through the mechanism of gynogenesis, because the sperm was occasionally recognized as heterologous sperm, just like that from other fish species owing to the significant genetic difference between clones A and D. The clone A-like individual should be suggested to form via androgenesis, because it has an entire nuclear genome from the paternal clone A and a mtDNA genome from the maternal clone D. In contrast to gynogenesis, the entire female nuclear genome of clone D might be extruded, and the offspring might be produced by two possible mechanisms, such as by ploidy doubling of clone A sperm through inhibiting the first mitotic division or by inclusion of clone A unreduced sperm into clone D ooplasm. If the latter is true, it is impossible to differentiate females and males in the androgenetic offspring (Figure 1) according to the XY sex determination mechanism[57], even though the direct evidence for existence of a few proportion of unreduced sperms have been obtained by flow cytometry[42] in the paternal clone A sperms (Figure 6). Therefore, we suggest that the clone A-like androgenetic individuals should be formed by the mechanism of ploidy doubling of clone A sperm through inhibiting the first mitotic division, and thereby the nucleo-cytoplasmic hybrids can differentiate into females and males.

Significantly, the selected nucleo-cytoplasmic hybrid female still maintains its gynogenetic ability, and thereby forms a nucleo-cytoplasmic clone with over several hundred millions of clonal individuals. In the past 20 years, we also obtained one allotetraploid clone with additional genome incorporation[58,59] and one supertriploid clone with subgenomic incorporation[13] in the gibel carp. Actually, unisexual clones were early synthesized in 1973 from *Poeciliopsis monacha-lucida*[60], and the stably inherited clone with minichromosome incorporation was also found from gynogenetic Amazon molly (*Poecilia formosa*)[12,61]. However, this androgenetic nucleo-cytoplasmic hybrid clone should be the first case in vertebrates, and might be related to the high polyploidy level and the diverse reproduction modes[8]. In invertebrate freshwater clams of the genus *Corbicula *that reproduce by androgenesis, ploidy changes including diploid, triploid, and tetraploid exist widely among different species and populations, and the higher ploidy levels have been suggested to be related to the origin of androgenetic reproduction[49,62]. In interspecific hybrids of Sicilian stick insects, various reproduction modes including parthenogenesis, hybridogenesis and androgenesis have been revealed to be associated with hybridization, polyploidy and karyotype re-diploidization, and these modes have been believed to be a noteworthy ability to overcome species-specific reproduction isolation mechanisms[48,63]. Similarly, various ploidy including triploid, supertriploid and tetraploid and different reproduction modes including gynogenesis, androgenesis and sexual reproduction also exist extensively in gibel carp. Therefore, gibel carp and even other unisexual vertebrates may also possess the ability for overcoming species-specific reproduction isolation mechanisms. In unisexual salamanders (genus *Ambystoma*), Bogart et al. revealed a new reproductive mode of kleptogenesis by which unisexual salamanders steal sperm genome from donors of normally bisexual species[33,34]. Perhaps, various ploidy levels and different reproduction modes are common characteristics for the unisexual animal complexes, regardless of whatever they are invertebrates or vertebrates, and it is these characteristics to result in rapid incorporation of nuclear genome, chromosome, chromosomal segment or mtDNA genome and to cause rapid genetic changes. Moreover, they achieve remarkable ecological success, even though some are rare and localized, and others are abundant [2]. For example, in comparison with the related sexual species *Carassius auratus*, polyploid gibel carp have wide geographic distribution and occupy more multiple niches from northern Europe to Asia[8].

Indeed, the coexisting dual modes of unisexual gynogenesis and sexual reproduction in gibel carp might be indicative case of a change in reproductive mode from complete unisexual reproduction towards sexual reproduction[8]. In green toads (*Bufo viridis*)[64,65] and water frogs (*Pelophylax esculentus*)[66] involving various ploidy levels, sexual reproduction triploids have been observed. Recently, a male-biased mutant family that contains 97.2% males was also found from the gynogenetic progeny of a gibel carp clone[37]. Therefore, various ploidy levels and highly diverse reproductive modes might be the causes for novel clone formation and clonal diversity in unisexual vertebrates, and also make them excellent models for the studies of evolutionary genetics and ecology, both theoretically and empirically. Additionally, as the nucleo-cytoplasmic hybrid clone escapes the genetic and developmental destruction caused by drastic treatments of irradiation and physical shocks in induced androgenesis[55] and nuclear transplantation[67,68], the novel finding will be of great significance for exploiting the genetic breeding approaches in gibel carp[69]. Of course, this finding of nucleo-cytoplasmic hybrid clone can help us to understand some reasons for clonal and genetic diversity production of unisexual animals, but it remains unknown whether similar clones also exist in natural populations of gibel carp. Therefore, further genetic resource survey will be required for elucidating the evolutionary mechanisms of polyploid gibel carp through the nuclear and cytoplasmic markers.

## Conclusions

In conclusion, we here identify a novel clone in polyploid gibel carp, and suggest that the novel clone might be a nucleo-cytoplasmic hybrid between the known clones A and D, because it originates from the offspring of gonochoristic sexual reproduction mating between clone D female and clone A male, and contains an entire nuclear genome from the paternal clone A and a mtDNA genome (cytoplasm) from the maternal clone D. It is suggested to arise via androgenesis by a mechanism of ploidy doubling of clone A sperm in clone D ooplasm through inhibiting the first mitotic division. Significantly, the selected nucleo-cytoplasmic hybrid female still maintains its gynogenetic ability, and thereby forms a novel clone with over several hundred millions of clonal individuals proliferated by 7 successive generations of gynogenesis. The androgenetic nucleo-cytoplasmic hybrid clone should be the first case in vertebrates, and might be related to the high polyploidy level and the diverse reproduction modes in gibel carp. Based on the present and previous findings, we discuss the association of rapid genetic changes and high genetic diversity with various ploidy levels and multiple reproduction modes in several unisexual and sexual complexes of vertebrates and even other invertebrates. Therefore, the novel finding will be of great significance not only for exploiting the genetic breeding approaches in gibel carp but also for the studies of evolutionary genetics and ecology in other unisexual animals.

## Competing interests

The authors declare that they have no competing interests.

## Authors' contributions

JFG, ZWW and LZ conceived and designed the experiments. HPZ contributed to the chromosome data, DW and ZWW contributed to the AFLP data, FFJ and ZWW contributed to the transferrin allele data, and WG and ZWW contributed to the microsatellite data. ZWW analyzed the data. JFG and ZWW wrote the paper. All authors have read and approved the final manuscript.

## References

[B1] Hubbs CL Hubbs LC Apparent Parthenogenesis in Nature, in a Form of Fish of Hybrid Origin Science 1932 76 628 630 10.1126/science.76.1983.628 17730035

[B2] Avise JC Clonality: The Genetics, Ecology, and Evolution of Sexual Abstinence in Vertebrates 2008 Oxford: Oxford University Press

[B3] Schlupp I The evolutionary ecology of gynogenesis Annu Rev Ecol Syst 2005 36 399 417 10.1146/annurev.ecolsys.36.102003.152629

[B4] Lampert KP Schartl M The origin and evolution of a unisexual hybrid: *Poecilia formosa* Philos Trans R Soc Lond B Biol Sci 2008 363 2901 2929 10.1098/rstb.2008.0040 18508756 PMC2606734

[B5] Lamatsch DK Stock M I Schon Sperm-dependent parthenogenesis and hybridogenesis in teleost fishes Lost Sex 2009 Springer Science+Business Media B.V 399 432 full_text

[B6] Lampert1 KP Schartl M A little bit is better than nothing: the incomplete parthenogenesis of salamanders, frogs and fish BMC Biology 2010 8 78 10.1186/1741-7007-8-78 20687905 PMC2914643

[B7] Zhou L Wang Y Gui JF Genetic evidence for gonochoristic reproduction in gynogenetic silver crucian carp (*Carassius auratus gibelio *Bloch) as revealed by RAPD assays J Mol Evol 2000 51 498 506 11080373 10.1007/s002390010113

[B8] Gui JF Zhou L Genetic basis and breeding application on clonal diversity and dual reproduction modes in polyploid *Carassius auratus gibelio* Sci China Life Sci 2010 53 409 415 10.1007/s11427-010-0092-6 20596906

[B9] Rylkova K Kalous L Slechtova V Bohlen J Many branches, one root: First evidence for a monophyly of the morphologically highly diverse goldfish (*Carassius auratus*) Aquaculture 2010 302 36 41 10.1016/j.aquaculture.2010.02.003

[B10] Cherfas NB Kirpichnikov VS Gynogenesis in fishes Genetic Bases of Fish Selection 1981 Berlin: Springer-Verlag 255 273

[B11] Jiang YG Liang SC Chen BD Yu HX Shan SX Yang DL Lin SE Shen GQ Biological effect of heterologous sperm on gynogenetic offspring in *Carassius auratus gibelio* Acta Hydrobiol Sin 1983 8 1 13

[B12] Schartl M Nanda I Schlupp I Wilde B Epplen JT Schmid M Parzefall J Incorporation of subgenomic amounts of DNA as compensation for mutational load in a gynogenetic fish Nature 1995 373 68 71 10.1038/373068a0

[B13] Yi MS Li YQ Liu JD Zhou L Yu QX Gui JF Molecular cytogenetic detection of paternal chromosome fragments in allogynogenetic gibel carp, *Carassius auratus gibelio *Bloch Chromosome Research 2003 11 665 71 10.1023/A:1025985625706 14606628

[B14] Zhou L Gui JF Karyotypic diversity in polyploid gibel carp, *Carassius auratus gibelio *Bloch Genetica 2002 115 223 232 10.1023/A:1020102409270 12403177

[B15] Zhu HP Ma DM Gui JF Triploid origin of the gibel carp as revealed by 5S rDNA localization and chromosome painting Chromosome Res 2006 14 767 776 10.1007/s10577-006-1083-0 17115331

[B16] Yang L Yang ST Wei XH Gui JF Genetic diversity among different clones of the gynogenetic silver crucian carp, *Carassius auratus gibelio*, revealed by transferrin and isozyme markers Biochem Genet 2001 39 214 225 10.1023/A:1010297426390 11530857

[B17] Zhou L Wang Y Gui JF Analysis of genetic heterogeneity among five gynogenetic clones of silver crucian carp, *Carassius auratus gibelio *Bloch, based on detection of RAPD molecular markers Cytogenet Cell Genet 2000 88 129 133 10.1159/000015506 10773687

[B18] Zhou L Wang Y Gui JF Molecular analysis of silver crucian carp (*Carassius auratus gibelio *Bloch) clones by SCAR markers Aquaculture 2001 201 219 228 10.1016/S0044-8486(01)00603-2

[B19] Guo W Gui JF Microsatellite marker isolation and cultured strain identification in *Carassius auratus gibelio* Aquaculture International 2008 16 497 510 10.1007/s10499-007-9161-7

[B20] Yang L Gui JF Positive selection on multiple antique allelic lineages of transferrin in the polyploid *Carassius auratus* Mol Biol Evol 2004 21 1264 1277 10.1093/molbev/msh121 15014154

[B21] Yang L Zhou L Gui JF Molecular basis of transferrin polymorphism in goldfish (*Carassius auratus*) Genetica 2004 121 303 313 10.1023/B:GENE.0000039855.55445.67 15521429

[B22] Li FB Gui JF Clonal diversity and genealogical relationships of gibel carp in four hatcheries Animal Genetics 2008 39 28 33 10.1111/j.1365-2052.2007.01671.x 18076744

[B23] Lampert KP Lamatsch DK Schories S Hopf A García de León FJ Schartl M Microsatellites for the gynogenetic Amazon molly, *Poecilia formosa*: useful tools for detection of mutation rate, ploidy determination and overall genetic diversity J Genet 2006 85 67 71 10.1007/BF02728973 16809843

[B24] Lampert KP Lamatsch DK Fischer P Epplen JT Nanda I Schartl M Automictic reproduction in interspecific hybrids of Poeciliid fish Current Biology 2007 17 1948 1953 10.1016/j.cub.2007.09.064 17980594

[B25] Angers B Schlosser IJ The origin of *Phoxinus eos-neogaeus *unisexual hybrids Mol Ecol 2007 16 4562 4571 10.1111/j.1365-294X.2007.03511.x 17892466

[B26] Quattro JM Avise JC Vrijenhoek RC Molecular evidence for multiple origins of hybridogenetic fish clones (Poeciliidae: *Poeciliopsis*) Genetics 1991 127 391 398 2004710 10.1093/genetics/127.2.391 PMC1204366

[B27] Quattro JM Avise JC Vrijenhoek RC An ancient clonal lineage in the fish genus *Poeciliopsis *(Atheriniformes: Poeciliidae) Proc Natl Acad Sci USA 1992 89 348 352 10.1073/pnas.89.1.348 11607248 PMC48234

[B28] Mateos M Vrijenhoek RC Ancient versus reticulate origin of a hemiclonal lineage Evolution 2002 56 985 92 12093033 10.1111/j.0014-3820.2002.tb01410.x

[B29] Hotz H Beerli P Spolsky C Mitochondrial DNA reveals formation of nonhybrid frogs by natural matings between hemiclonal hybrids Mol Biol Evol 1992 9 610 620 1352842 10.1093/oxfordjournals.molbev.a040744

[B30] Spolsky CM Phillips CA Uzzell T Antiquity of clonal salamander lineages revealed by mitochondrial DNA Nature 1992 356 706 708 10.1038/356706a0 1570013

[B31] Hedges SB Bogard JP Maxson LR Ancestry of unisexual salamanders Nature 1992 356 708 710 10.1038/356708a0 1570014

[B32] Robertson AV Ramsden C Niedzwiecki J Fu J Bogart JP An unexpected recent ancestor of unisexual Ambystoma Mol Ecol 2006 15 3339 3351 10.1111/j.1365-294X.2006.03005.x 16968274

[B33] Bogart JP Bi K Fu J Noble D Niedzwiecki J Unisexual salamanders (genus *Ambystoma*) present a new reproductive mode for eukaryotes Genome 2007 50 119 136 10.1139/G06-152 17546077

[B34] Bogart JP Bartoszek J Noble DW Bi K Sex in unisexual salamanders: discovery of a new sperm donor with ancient affinities Heredity 2009 103 483 93 10.1038/hdy.2009.83 19639004

[B35] Bi K Bogart JP Time and time again: unisexual salamanders (genus *Ambystoma*) are the oldest unisexual vertebrates BMC Evol Biol 2010 10 238 10.1186/1471-2148-10-238 20682056 PMC3020632

[B36] Badaeva TN Malysheva DN Korchagin VI Ryskov AP Genetic variation and de novo mutations in the parthenogenetic Caucasian rock lizard *Darevskia unisexualis* PLoS ONE 2008 3 7 e2730 10.1371/journal.pone.0002730 18648496 PMC2447159

[B37] Wang D Mao HL Peng JX Li XY Zhou L Gui JF Discovery of a male-biased mutant family and identification of a male-specific SCAR marker in gynogenetic gibel carp *Carassius auratus gibelio* Prog in Nat Sci 2009 19 1537 1544 10.1016/j.pnsc.2009.04.008

[B38] Gui JF Wu C, Gui JF Fish developmental genetics and artificial propagation Fish Genetics and Breeding Engineering 1999 Shanghai Scientific and Technical, Shanghai 41 62

[B39] Wei WH Wells RA Baldini A Reeders ST Karyotyping of *Brassica napus *L. based on *Cot-1 *DNA Banding by Fluorescence in situ Hybridization J Integr Plant Biol 2005 47 1479 1484 10.1111/j.1744-7909.2005.00186.x

[B40] Wang D Mao HL Chen HX Liu HQ Gui JF Isolation of Y- and X-linked SCAR markers in yellow catfish and application in the production of all-male populations Animal Genetics 2009 40 978 981 10.1111/j.1365-2052.2009.01941.x 19694649

[B41] Sun YD Zhang C Liu SJ Duan W Liu Y Induced interspecific androgenesis using diploid sperm from allotetraploid hybrids of common carp × red crucian carp Aquaculture 2007 264 47 53 10.1016/j.aquaculture.2006.07.004

[B42] Wei WH Zhang J Zhang YB Zhou L Gui JF Genetic heterogeneity and ploidy level analysis among different gynogenetic clones of polyploid gibel carp Cytometry 2003 56 46 52 10.1002/cyto.a.10077 14566938

[B43] Excoffier L Laval G Schneider S Arlequin ver. 3.0: An integrated software package for population genetics data analysis Evolutionary Bioinformatics Online 2005 1 47 50 19325852 PMC2658868

[B44] Thompson JD Gibson TJ Plewniak F Jeanmougin F Higgins DG The ClustalX windows interface: flexible strategies for multiple sequence alignment aided by quality analysis tools Nucleic Acids Research 1997 24 4876 4882 10.1093/nar/25.24.4876 PMC147148 9396791

[B45] Tamura K Dudley J Nei M Kumar S MEGA4: Molecular evolutionary genetics analysis (MEGA) software version 4.0 Mol Biol Evol 2007 24 1596 1599 10.1093/molbev/msm092 17488738

[B46] Johansen SD Coucheron DH Andreassen M Karlsen BO Furmanek T Jørgensen TE Emblem A Breines R Nordeide JT Moum T Nederbragt AJ Stenseth NC Jakobsen KS Large-scale sequence analyses of Atlantic cod New Biotechnology 2009 25 263 271 10.1016/j.nbt.2009.03.014 19491044

[B47] Mantovani B Scali V Hybridogenesis and androgenesis in the stick-insect *Bacillus rossius-grandii benazzi *(Insecta Phasmatodea) Evolution 1992 46 783 796 10.2307/2409646 28568678

[B48] Milani L Ghiselli F Pellecchia M Scali V Passamonti M Reticulate evolution in stick insects: the case of Clonopsis (Insecta Phasmida) BMC Evol Biol 2010 10 258 10.1186/1471-2148-10-258 20738851 PMC2936309

[B49] Komaru A Kawagishi T Konishi K Cytological evidence of spontaneous androgenesis in the freshwater clam Corbicula leana Prime Dev Genes Evol 1998 208 46 50 10.1007/s004270050152 9518524

[B50] Hedtke SM Stanger-Hall K Baker RJ Hillis DM All-male asexuality: origin and maintenance of androgenesis in the asian clam *corbicula* Evolution 2008 65 1119 1136 10.1111/j.1558-5646.2008.00344.x 18266987

[B51] Thorgaard GH Scheerer PD Hershberger WK Myers JM Androgenetic rainbow trout produced using sperm from tetraploid males show improved survival Aquaculture 1990 85 215 221 10.1016/0044-8486(90)90021-E

[B52] Bongers ABJ Abarca BJ Doulabi ZB Eding EH Komen J Richter CJJ Maternal influence on development of androgenetic clones of common carp, *Cyprinus carpio *L Aquaculture 1995 137 139 147 10.1016/0044-8486(96)83544-7

[B53] Arai K Ikeno M Suzuki R Production of androgenetic diploid loach *Misgurnus anguillicaudatus *using spermatozoa of natural tetraploids Aquaculture 1995 137 131 138 10.1016/0044-8486(95)01106-4

[B54] Nam YK Cho YS Kim DS Isogenic transgenic homozygous fish induced by artificial parthenogenesis Transgenic Res 2000 9 463 469 10.1023/A:1026596422225 11206975

[B55] Grunina AS Recoubratsky AV Induced androgenesis in fish: obtaining viable nucleocytoplasmic hybrids Russian Journal of Developmental Biology 2005 36 208 217 10.1007/s11174-005-0035-5 16208936

[B56] Komen H Thorgaard GH Androgenesis, gynogenesis and the production of clones in fishes: A review Aquaculture 2007 269 150 173 10.1016/j.aquaculture.2007.05.009

[B57] Devlin RH Nagahama Y Sex determination and sex differentiation in fish: an overview of genetic, physiological, and environmental influences Aquaculture 2002 208 191 364 10.1016/S0044-8486(02)00057-1

[B58] Gui JF Liang SC Zhu LF Jiang YG Discovery of multiple tetraploids in artificially propagated populations of allogynogenetic silver crucian carp and their breeding potentialities Chinese Science Bulletin 1993 38 327 331

[B59] Zhu HP Gui JF Identification of genome organization in the unusual allotetraploid form of *Carassius auratus gibelio* Aquaculture 2007 265 109 117 10.1016/j.aquaculture.2006.10.026

[B60] Schultz RJ Unisexual fish: laboratory synthesis of a "species Science 1973 179 180 181 10.1126/science.179.4069.180 4682248

[B61] Nanda I Schlupp I Lamatsch DK Lampert KP Schmid M Schartl M Stable inheritance of host species-derived microchromosomes in the gynogenetic fish, Poecilia formosa Genetics 2007 177 917 926 10.1534/genetics.107.076893 17720916 PMC2034654

[B62] Qiu A Shi A Komaru A Yellow and brown shell color morphs of *Corbicula fluminea *(Bivalvia: Corbiculidae) from Sichuan Province, China, are triploids and tetraploids Journal of Shellfish Research 2001 20 323 328

[B63] Milani L Scali V Passamonti M The Clonopsis gallica puzzle: Mendelian species, polyploid parthenogens with karyotype re-diploidization and clonal androgens in Moroccan stick insects (Phasmida) J Zool Syst Evol Res 2009 47 132 140 10.1111/j.1439-0469.2008.00489.x

[B64] Stöck M Lamatsch DK Steinlein C Epplen JT Grosse WR Hock R Klapperstück T Lampert KP Scheer U Schmid M Schartl M A bisexually reproducing all-triploid vertebrate Nat Genet 2002 30 325 328 11836500 10.1038/ng839

[B65] Stöck M Ustinova J Lamatsch DK Schartl M Perrin N Moritz C A vertebrate reproductive system involving three ploidy levels: hybrid origin of triploids in a contact zone of diploid and tetraploid palearctic green toads (*Bufo viridis *subgroup) Evolution 2010 64 944 959 19863582 10.1111/j.1558-5646.2009.00876.x

[B66] Christiansen DG Reyer HU From clonal to sexual hybrids: genetic recombination via triploids in all-hybrid populations of water frogs Evolution 2009 63 1754 1768 10.1111/j.1558-5646.2009.00673.x 19245393

[B67] Sun YH Chen SP Wang YP Hu W Zhu ZY Cytoplasmic impact on cross-genus cloned fish derived from transgenic common carp (*Cyprinus carpio*) nuclei and goldfish (*Carassius auratus*) Biology of Reproduction 2005 72 510 515 10.1095/biolreprod.104.031302 15469998

[B68] Fujimoto T Saito T Sakao S Arai K Yamaha E Developmental potential of embryonic cells in a nucleocytoplasmic hybrid formed using a goldfish haploid nucleus and loach egg cytoplasm Int J Dev Biol 2010 54 827 835 10.1387/ijdb.092896tf 20336611

[B69] Gui JF Genetic Basis and Artificial Control of Sexuality and Reproduction in Fish 2007 Beijing: Science Press

